# Interventions to promote medication adherence for chronic diseases in India: a systematic review

**DOI:** 10.3389/fpubh.2023.1194919

**Published:** 2023-06-16

**Authors:** Abraham Tolley, Refaat Hassan, Rohan Sanghera, Kirpal Grewal, Ruige Kong, Baani Sodhi, Saurav Basu

**Affiliations:** ^1^University of Cambridge, Cambridge, United Kingdom; ^2^Indian Institute of Public Health-Delhi, Gurugram, India

**Keywords:** India, systematic review, interventions, medication adherence, non-communicable diseases

## Abstract

**Introduction:**

Cost-effective interventions that improve medication adherence are urgently needed to address the epidemic of non-communicable diseases (NCDs) in India. However, in low- and middle-income countries like India, there is a lack of analysis evaluating the effectiveness of adherence improving strategies. We conducted the first systematic review evaluating interventions aimed at improving medication adherence for chronic diseases in India.

**Methods:**

A systematic search on MEDLINE, Web of Science, Scopus, and Google Scholar was conducted. Based on a PRISMA-compliant, pre-defined methodology, randomized control trials were included which: involved subjects with NCDs; were located in India; used any intervention with the aim of improving medication adherence; and measured adherence as a primary or secondary outcome.

**Results:**

The search strategy yielded 1,552 unique articles of which 22 met inclusion criteria. Interventions assessed by these studies included education-based interventions (*n* = 12), combinations of education-based interventions with regular follow up (*n* = 4), and technology-based interventions (*n* = 2). Non-communicable diseases evaluated commonly were respiratory disease (*n* = 3), type 2 diabetes (*n* = 6), cardiovascular disease (*n* = 8) and depression (*n* = 2).

**Conclusions:**

Although the vast majority of primary studies supporting the conclusions were of mixed methodological quality, patient education by CHWs and pharmacists represent promising interventions to improve medication adherence, with further benefits from regular follow-up. There is need for systematic evaluation of these interventions with high quality RCTs and their implementation as part of wider health policy.

**Systematic review registration:**

https://www.crd.york.ac.uk/prospero/display_record.php?ID=CRD42022345636, identifier: CRD42022345636.

## Introduction

Non-communicable diseases (NCDs) represent leading causes of morbidity and mortality worldwide, with a disproportionate burden in low- and middle-income countries (LMICs) (1). India is currently undergoing a significant epidemiological, demographic, and socioeconomic transition which is driving an epidemic of NCDs—which now accounts for around 4.7 million deaths per year and 226.8 million disability-adjusted life years (2).

Treatment of NCDs usually requires long-term medication adherence to maintain optimal health outcomes, prevent the onset and progression of complications, and improve the quality of life (3, 4). However, the World Health Organization (WHO) estimates 50% of patients in developed countries do not take their medications as prescribed (5). This figure has been reported to be lower in LMICs like India for a range of NCDs (6–9). In India, several barriers to adherence have been identified across the WHO's five dimensions of medication adherence (5, 10). This includes patient-related factors such as poor understanding of illness and treatment owing to low rates of health literacy (11), healthcare-related factors such as healthcare accessibility (11), medication-related factors such as medication affordability secondary to financial constraints (12), condition-related factors such as the development of complications (13), and socioeconomic factors including socioeconomic status, and existing untreated substance abuse disorder (14). In particular, patient-related factors appear most amenable to interventions that aim to improve medication adherence (15, 16).

There is growing evidence showing the benefits of medication adherence improving interventions on NCDs control (17, 18). However, most of the evidence for medication-adherence improving strategies is based on western populations (19); does not distinguish between countries of origin (20), or excludes studies from LMICs due to differences in healthcare systems (21). Existing evidence on adherence promoting interventions in LMICs tends to focus on certain diseases across LMICs without stratifying country-level effects (22, 23) or is specific for communicable diseases, such as HIV (24, 25), where distinct factors such as complex regimens, greater perceived risk, and stigma may influence adherence, compared to NCDs (25, 26).

Given the increasingly aging population in India and the concomitant growing burden of NCDs, low medication adherence imposes a significant healthcare, and financial cost (27, 28). As low medication adherence is partly driven by behavioral factors in India (10–14), there is potential for effective medication-adherence improving interventions to improve health outcomes, which is also one of WHO's key current priorities (5, 29). There is need for effective medication adherence-improving interventions based on systematically analyzed evidence. However, to our knowledge, there are no systematic reviews evaluating adherence-promoting interventions in individual LMICs, such as India, which focus on a variety of NCDs. Furthermore, country-specific reviews are important to ensure generalizability of primary studies and tailored large-scale public health interventions to improve real-world outcomes.

This systematic review sought to review the evidence for interventions that promote medication adherence in India for patients with NCDs and provide a qualitative synthesis of results. We aim to allow policy makers and stakeholders to make evidence-based decisions on which adherence promoting interventions are effective, scalable, replicable, generalizable, and cost-effective, disaggregating findings by intervention and NCD. We also aimed to review strategies for measuring medication adherence and provide recommendations to inform design of future studies.

## Methods

### Protocol registration

The aim of this study was to conduct a systematic review evaluating the efficacy of interventions aimed at improving medication adherence for chronic diseases in India. The study protocol was registered on PROSPERO (Registration ID: CRD42022345636). A preliminary scoping search was performed to refine search criteria and identify outcome measures. This study was undertaken according to the Preferred Reporting Items for Systematic Reviews and Meta-Analyses (PRISMA) framework (30). A PRISMA (2020) checklist is shown in the Supplementary material.

### Eligibility criteria

The study design was developed using the PICO (participant, interventions, comparisons, outcomes) framework (31).

### Participant criteria

Only studies which investigated adherence interventions in India, in outpatient and community populations with at least one eligible non-communicable chronic disease, were considered. NCDs appropriate for inclusion were identified previously in the scoping search and based on those surveyed in the recent SAGE-2 report in India: stroke, angina pectoris, diabetes mellitus, asthma, depression, hypertension, and chronic lung disease (32). We excluded communicable diseases, such as tuberculosis and HIV, as interventions to improve adherence to medications for these diseases are better characterized (24, 33–37). We also excluded arthritis, as regular medication use is not the mainstay of treatment (38, 39).

### Intervention & outcome criteria

Only studies which tested an intervention to improve adherence in patients taking regular medications, either as a primary or secondary outcome, were considered. Examples of eligible interventions were patient education, streamlined medication regimens, and electronic reminders. Studies only investigating interventions targeted at improving medication adherence within a hospital setting were excluded. Adherence outcomes included subjective data based on patient self-reporting and objective measures, such as pill counts. Studies which measured objective clinical parameters and disease outcomes as a proxy for adherence were also included if adherence to treatment was explicitly stated as a study aim.

### Search strategy

A comprehensive search strategy, based on the above criteria and the scoping review, was devised. The search strategy was conducted on Ovid Medline, Web of Science, Scopus, and Google Scholar. No restrictions were placed on language, date of publication or geographical region. The full search criteria can be found in Supplementary material. Medical subject headings (MeSH), free-text terms, and Boolean logic were used where available. Searches were run on 20/7/2022. Given the comprehensive search strategy, references of included articles were not checked.

### Study selection

The studies retrieved by the search strategy, along with study information and abstract text, were imported into *Zotero* and then *Mendeley* for de-duplication (40). A 1,264 unique articles were split amongst reviewers, with each title and abstract screened independently by two reviewers using *Rayyan AI* (41). Conflicts between the two reviewers were resolved by a third independent reviewer. To ensure the inclusion and exclusion criteria were applied consistently amongst all reviewers, all reviewers screened 50 studies first and any disagreements were discussed by all reviewers until a consensus was reached. Full texts of included studies were screened independently by two reviewers; a third independent reviewer resolved any disagreements between the two original reviewers. For reports where full text was unable to be retrieved, authors were contacted to request their manuscripts—if they failed to respond within 2 months, studies were excluded.

### Study criteria

Only randomized control trials were included in qualitative synthesis to ensure conclusions were informed by the best evidence available. Inclusion and exclusion criteria are shown in Table 1.

**Table 1:** Inclusion and exclusion criteria.

**Inclusion criteria**	**Exclusion criteria**
Peer-reviewed primary article in English	Secondary data analysis, retracted article or a non-peer reviewed article
Subjects are located in India	Study includes some subjects from India but India-specific data is inaccessible
Subjects have an established non-communicable chronic disease	Study focuses on osteoarthritis or communicable diseases e.g., HIV or tuberculosis
Study design is a randomized control trial	Study design is not a randomized control trial
Subjects are exposed to an intervention with the primary or secondary intended effect of increasing medication adherence	Study was not a primary article reporting results of a trial relating to medication adherence^*^
Subjects are located within the community when taking medication (subjects may be recruited from a hospital)	Study population are hospital inpatients

^*^Several included articles reported the results from the same primary study. Only the primary article(s) reporting results relating to medication adherence were included.

### Data extraction and management

Data extraction was conducted via Google Sheets using a pre-specified template, using a list of features identified during the scoping review. One reviewer performed data extraction for each study, and this was subsequently checked by a second reviewer. The different domains of data extracted are reported in Supplementary material.

### Quality assessment

The Cochrane Risk-of-Bias (RoB) 2 tool for randomized control trials and for cluster randomized control trials were used to assess methodological quality (42). RoB assessment was conducted independently by two reviewers for each article, and any disagreements across domains were resolved by a third reviewer. Results were synthesized and formatted using R Studio (43). When multiple papers reported different adherence-related outcomes from the same original study dataset, study weights were adjusted accordingly to prevent double-counting.

## Results

**Figure 1:**
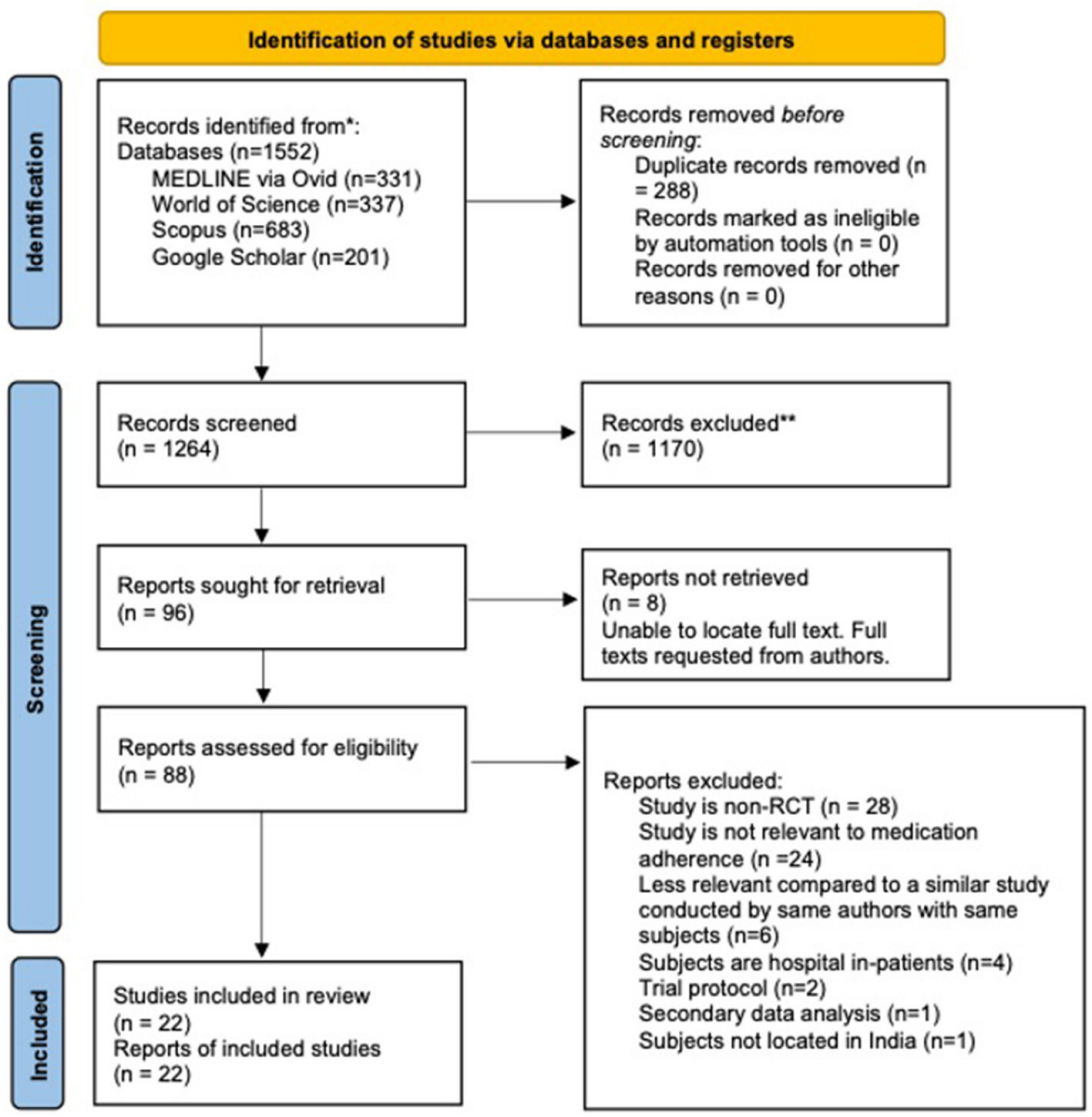
PRISMA (2020) flowchart detailing information flow throughout the review process.

A total of 1552 studies were identified from literature searches, of which 288 were removed as duplicates. A further 1,170 were excluded during title and abstract screening. A total of 96 full texts were screened for eligibility, of which 22 were included in this review. A PRISMA flow diagram depicting information flow through different phases of the systematic review is shown in Figure 1.

### Overview of interventions

We identified 22 RCTs which met the inclusion criteria (44–65). Of these 22 studies, 12 investigated the effects of patient education (mean length of follow-up = 6 months); two studies investigated the use of electronic reminder-based systems (mean length of follow-up = 9 months); two studies investigated psychological-based approaches (mean length of follow-up = 3.5 months), single studies investigated medication regimen changes (length of follow-up = 12 months) and improved clinical practitioner competency (length of follow-up = 5 months), and four RCTs used multiple interventions (mean length of follow-up = 12 months). Studies all evaluated adult patients (with the exception of Grover et al. which focused on pediatric patients) (44) with a mean age of patients between 50–65 (with Raj et al. focussing on those over 60 exclusively) (60) of both sexes with Pradeep et al. focussing on only female patients (49). Studies collected their data between 2006 and 2019 where timeframes were given (Ponnusankar et al. was published in 2004) (45). An overview of all included studies can be found in Table 2 below. Many studies had multiple outcome measures; those most relevant to adherence and corresponding clinical outcomes are reported in Table 2.

**Table 2:** Summary of individual trials, by intervention strategy.

**Source (clusters marked)**	**Population (adult unless otherwise specified)**	**Location(s) and time of study**	**Intervention details & sample demographics**	**Comparison group & sample demographics**	**Outcome measure(s)**	**Effect size & time scale**
**Patient education–by pharmacists**						
Grover et al. (44)	Children aged 7-12 years old with asthma along with their parents	Delhi July–December 2012	Educational programme - presentation, workbook and activities delivered by 2 pharmacists. 24 pairs of parent and child Mean age: 10.2 67% Male	Usual care 16 pairs of parent and child Mean age: 10.4 63% Male	BMQ Questionnaire Asthma control score via asthma control questionnaire	Mean BMQ score improved from 1.5 to 0.26 in the intervention group vs. no change from 1.4 in the control group after 6 months. Significant improvement in asthma control score (*p < * 0.001) in intervention vs. baseline after 6 months
Srirama et al. (65)	T2DM	Tamil Nadu (Coimbatore) May 2009-December 2009	Pharmaceutical care by pharmacist including medication counseling and leaflets 60 subjects Mean age: 53.4 50% Male	Usual care 60 subjects Mean age: 58.0 50% Male	HbA1c, FBG, quality of life	Significant Improvement in HbA1c, quality of life and FBG in intervention after 8 months (*p < * 0.01) vs. no significant change in control after 8 months
Renuga et al. (59)	T2DM	South India Timeframe not stated	Continuous counseling at baseline,1,2 and at 3 months follow-up (30 min per session) by clinical pharmacists with provision of patient education leaflets 200 subjects Mean Age: 57.8 22% Male	Counseling at baseline and at 3 months follow-up and provided patient education leaflets 200 subjects Mean Age: 57.6 11% Male	MAQ questionnaire FBS	Significant improvement in mean adherence in both groups after 3 months (*p < * 0.001) & Increase in adherence score was significantly higher in intervention than control group (*p < * 0.001) at 3 months Significant reduction in mean fasting blood sugar in both groups but statistically significantly higher in intervention group (*p < * 0.001) at 3 months
Simon et al. (57)	T2DM	Multicentre (specific locations not specified) Trial registered February 2019. Timeframe not stated	Pharmacist led verbal counseling including adherence (20 min, single session) with patient information leaflet 46 subjects Mean Age: 56.8 54% Male	Usual care 47 subjects Mean Age: 56.8 63.8% Male	MARS questionnaire HbA1c	Significant Improvement in adherence in intervention vs. control (*p < * 0.001) at 6 months Significant improvement in HbA1c (*p < * 0.001) in intervention vs. control at 6 months
Sundarajan et al. (54)	Post-MI	Tamil Nadu November 2017-April 2018	Pharmacist education including adherence (30 min, single session). Patient information leaflets were explained and provided during discharge 75 subjects Mean Age: 56.3 76% Male	Usual care 75 subjects Mean Age: 53.9 86.7% Male	MARS questionnaire Clinical parameters e.g., BP, FBS and total cholesterol	Significant improvement in medication adherence (*p =* 0.0001) at 6 months Significant improvement in clinical parameters e.g., SBP, DBP, FBS, total cholesterol (*p =* 0.003,*p =* 0.007,*p =* 0.04,*p < * 0.001 respectively) in intervention vs. control at 6 months
Sathvik et al. (48)	Hypertension	Karnataka Timeframe not stated	Pharmacist education regarding prescribed medications at baseline, 15th, 30th and 45th day 75 subjects 17.3% aged 41–50 40% aged 51–60 18.7% aged 61–70 53.3% Male	Usual care 75 subjects 20% aged 41–50 36% aged 51–60 25.3% aged 61–70 41.3% Male	BMQ questionnaire (broken down by belief, recall, access and regimen scores)	Significant improvement in, belief (*p =* 0.03) and recall (*p =* 0.05) BMQ scores of intervention vs. control at 2 months follow-up but no significant difference in regimen and access scores.
Ponnusankar et al. (45)	Chronic conditions like hypertension, T2DM, CVD, and asthma	South India Timeframe not stated	Pharmacist-led counseling on disease, medication and dosage (single session) 30 subjects 6.7% aged ≤ 40 66.% aged 41–60 26.6% aged ≥61 63.3% Male	Usual care 60 subjects 8.3% aged ≤ 40 63.3% aged 41–60 28.3% aged ≥61 51.7% male	Pill count method to calculate percentage compliance Self-assessment form	Intervention group adherence was 92.24 vs. 84.71% in the control group at 2 months follow-up. 75% of patients in intervention rated themselves as always compliant vs. 66.6% in the control group
**Patient education–by CHW**						
Gamage et al. (63) Cluster	Hypertension	3 Regions - Kerala (Trivandrum region), Andhra Pradesh (Rishi Valley and Western Godavari) November 2015-September 2016 *(different start dates in different regions)*	CHWs monitored BP, provided education about hypertension including importance of adherence to medication every 2 weeks for 3 months 637 subjects Mean age: 56.6 41.3% Male	Usual care 1097 subjects Mean age: 56.9 42.1% Male	Use of antihypertensive medication via interview Control of hypertension (number with B*P < * 140/90mmHg)	No effect seen with intervention Significant increase in hypertension control in intervention vs. control (*p =* 0.001) after 3 months
Pradeep et al. (49) Cluster	Women with Major Depressive Disorder	Rural Bangalore August 2006–September 2009	CHWs visited patients providing education and encouraging treatment adherence (visits occurred twice a month). CHWs also visited patients who discontinued medication 138 women 26.8% 26–35 27.5% aged 36–45 26.1% aged 46–55 0% Male	Usual care where patient was encouraged by physician during follow-up consultations 122 women 24.6% aged 26–35 35.2% aged 36–45 24.6% aged 46–55 0% Male	Total number of weeks taking antidepressants Hamilton depression rating scale	Weeks of treatment adherence was significantly greater in intervention vs. control group (*p < * 0.01) at 6 months No significant difference in severity of depression or QoL between intervention and control group although both groups improved compared to baseline at 6 months
Joshi et al. (61) Cluster	Intermediate-to-high risk of CVD	3 rural regions, not specified August 2011-February 2012	CHWs monitored risk factors, ascertained and reinforced adherence during 6 household visits over 12 months, every 2 months 1650 subjects Mean age: 61.7 % Male not given	Usual care 1611 subjects Mean age: 61.7 % Male not given	Proportion of consumed and prescribed number of pills SBP	Adherence to antihypertensive drugs was significantly greater in intervention vs. control (*p =* 0.001) at 12 months. No significant difference between SBP at 12 months (*p =* 0.18) though both groups saw a significant reduction compared to baseline (*p < * 0.01)
Xavier et al. (64)	Acute coronary syndrome	New Delhi, Jaipur, Lucknow, Bhopal, Nagpur, Wardha, Hyderabad, Secunderabad, Pune, Shivamogga, Bangalore, Chennai, Coimbatore, Kottayam August 2011–June 2012	CHWs delivered 6 sessions where discussed strategies for adherence, assessed and reinforced the need for adherence and discussed lifestyle measures (4 visits in hospitals - at discharge and in outpatient clinics, 2 home visits over one year) 404 subjects Mean age: 55.9 82% Male	Usual care 401 subjects Mean age: 56.9 83% Male	Composite medication adherence scale (≥80% score counted as adherent) Change in BP, BMI, HR, cholesterol	Significantly increased adherence in the intervention group vs. control (*p =* 0.006) after 1 year Significantly lower SBP (*p =* 0.002) and BMI (*p < * 0.0001) in intervention vs. control. No significant change in HR, DBP or cholesterol after 1 year.
**Patient Education - by multidisciplinary team**						
Sadeghian et al. (58)	T2DM	Delhi March 2010-May 2013	Educational self management programme by a multidisciplinary medical team. Group education. 2x2hr sessions in small groups including information on taking medication. 134 subjects 29.6% aged ≤ 40 39.5% aged 41–50 30.9% aged 51–60 42.1% Male	Routine treatment 123 subjects 20.1% aged ≤ 40 42.2% aged 41–50 37.5% aged 51–60 36.4% Male	HbA1c	Significantly greater fall in HbA1c in intervention vs. control (*p =* 0.001) after 6 months.
**Training Practitioners**						
Sylaja et al. (46) Cluster	Stroke / TIA survivors	Kerala (Thiruvananthapuram) December 2017-December 2018	Formal training programme for Community Health Workers including importance of educating on medication adherence. 114 subjects Mean age: 59.8 69.3% Male	Community health workers who did not receive additional training. 120 subjects Mean age: 59.4 71.7% Male	Number of patients advised to adhere to medications Control of hypertension (systolic BP) and diabetes (FBS)	Significant increase in intervention vs. control (*p < * 0.001) at 6 months No significant differences at 6 months
**Combining education from non-physicians with regular follow-up**						
Abdulsalim et al. (56)^*^	COPD	Manipal Recruitment March 2012-June 2013 & 2 years follow-up	Pharmacist education (single session, 15–20 minutes) placing emphasis on adherence, smoking cessation, exercise, inhaler use and need for timely follow-up (*n =* 130). Further follow-up by monthly phone calls ensuring adherence. Patient information leaflets provided. 104 subjects Mean age: 60.6 96.9% Male	Usual care 98 subjects Mean age: 61.1 94.4% Male	MAQ questionnaire	Significant improvement in medication adherence in intervention vs. control at all follow-up time points up to 2 years (*p < * 0.001)
Suhaj et al. (62)^*^	COPD	Manipal Patients screened March 2012-June 2013 and f	Clinical pharmacist led counseling (one-on-one, 15–20 minutes) and patient information leaflets. Patients received monthly telephones for medication adherence. During follow-up (every 6 months) patients were provided further motivation for adherence. 104 subjects Mean age: 60.6 96.9% Male	Usual care with 6 months follow-up. 98 patients Mean age: 61.1 94.4% Male	Health-related quality of life (HrQOL) via St. George's Respiratory Questionnaire	Significant improvement in intervention vs. control (*p < * 0.001) after 2 years
Raj et al. (60)	NCDs among (age > 60) including T2DM, hypertension, dyslipidemia and coronary artery disease among	Karnataka (Bangalore) January 2016 to December 2017	Education and tailored advice delivered by trial investigators, medication diary and telephone reminders. 25 subjects Mean age: 69.1 48% male	Usual Care 25 subjects Mean age: 69.2 60% male	Change in reported pill counts Clinical parameters–BP, blood glucose and serum lipids	Significantly improved reported pill counts in intervention vs. control at 3 months (*p =* 0.007) and 6 months (*p =* 0.003) No significant differences between intervention and control
Sheilini et al. (52)	Hypertension	Manipal July 2013 to February 2017	Nurse led individualized teaching session with information leaflets, focussing on medication adherence. Combined with weekly medication-reminder boxes and telephone reminder for follow-up 80 subjects 42.1% aged 60 to 70 17.8% aged >70 42.2% male	Usual care 80 subjects 38.8% aged 60 to 70 19.4% aged >70 53.3% male	MAS Change in SBP and DBP	Significant improvement in medication adherence in intervention vs. control group (*p < * 0.001)at 6 months No improvement in SBP or DBP in intervention vs. control (*p* > 0.05) at 6 months
**Technology based interventions**						
Kleinman et al. (47)	T2DM	Ahmedabad, Mumbai, Chennai March 2015 to January 2016	Mobile Health: diabetes management smartphone app and Web portal 44 subjects Mean age: 48.8 81.8% male	Usual care 47 subjects Mean age: 48 58.7% male	Self-reported medication adherence HbA1c	Significant improvement of medication adherence in intervention vs. control (*p* = 0.03) after 6 months Significant improvement of HbA1c in intervention vs. control (*p* = 0.02) after 6 months
Shetty et al. (51)	T2DM	Chennai Time of study not stated	Text messages to reinforce adherence every 3 days to follow dietary modification regime, physical activity, and drug schedules 110 subjects Mean age: 50.1 % male not reported	Usual care 105 subjects Mean age: 50.5 % male not reported	Validated questionnaire to assess adherence. BMI, Fasting plasma glucose, HbA1c, total cholesterol, LDL	Drug prescriptions were followed satisfactorily by both intervention and control groups. Significant decrease in fasting plasma glucose (*p < * 0.002) and LDL (*p < * 0.02) in intervention vs. control at 1 year. No significant difference in HbA1c, BMI or total cholesterol
**Fixed dose combinations**						
Thom et al. (50)	CVD or at risk of CVD	Bikaner, Delhi, Lucknow, Ludhiana, Jaipur, Chandigarh, Trivandrum, Hyderabad, Chennai, Pune, Mysore, Mumbai (July 2010 to July 2012)	Fixed-dose combination-based strategy 501 subjects Mean age: 62.1 81.5% male Demographic data for subjects in India not reported, overall data (India & Europe) provided	Usual care 499 subjects Mean age: 61.6 82.3% male Demographic data for subjects in India not reported, overall data (India & Europe) provided	Self-reported medication adherence Change in systolic BP and LDL	Significantly improved adherence in intervention vs. control (*p < * 0.001) at 15 months Significantly reduced SBP and LDL in intervention vs. control (*p < * 0.001) at 15 months
Valsaraj et al. (53)	Chronic Kidney Disease undergoing dialysis	Karnataka January 2013 to February 2014	Cognitive Behavioral Therapy (10 individual 50 minute sessions on weekly basis delivered by trained therapist) 33 subjects 67% aged 43 to 65 70% male	Non directive counseling on importance of adherence, with same number/ duration of sessions 34 subjects 66% aged 43 to 65 71% male	Haemodialyssi adherence scale including drug adherence subscale based on questionnaire Change in systolic BP, diastolic BP, Hb and inter-dialysis weight gain	Drug adherence score significantly increased in intervention vs. control (*p =* 0.001) after 6 months Significant decrease in systolic BP (*p =* 0.001) diastolic BP (*p =* 0.001) and inter-dialysis weight gain (*p =* 0.001), and significant increase in Hb (*p =* 0.001) in intervention vs. control after 6 months.
Pillai et al. (55) Cluster	Depression	Goa April 2007 to September 2009	Collaborative stepped care management model including psychoeducation, interpersonal psychotherapy, and collaborative case management 1360 subjects 20.4% aged 30–39 26.2% aged 40–49 23.8% 60 years and over 17.6% male Demographic data not split for intervention and control	Enhanced usual care (treatments of choice could be initiated) 1436 subjects 20.4% aged 30–39 26.2% aged 40–49, 23.8% 60 years and over, 17.6% male Demographic data not split for intervention and control	Self-report of antidepressant adherence for 1 month of those who received an antidepressant prescription	66.8% adherent with intervention vs. 31% in usual care (OR 6.10) After 1 month Significantly higher proportion with intervention completed at least 90 days of treatment vs. usual care *P values not stated*

BP; blood pressure; BMI, body mass index; CHW, community health worker; CVD, cardiovascular disease; BMQ, brief medication questionnaire; DBP, diastolic blood pressure; FBS, fasting blood sugar; HR, heart rate; LDL, low density lipoprotein; MARS, medication adherence rating scale; MAQ, Morisky Adherence Questionnaire; MI, myocardial infarction; SBP, systolic blood pressure; T2DM, type 2 diabetes mellitus. ^*^trials based on same original data set. Average age given where possible–otherwise percentage of patients in up to three age bands reported.

### Overview of adherence measurements

Adherence can be measured directly, through objective parameters such as pill counts and subjective parameters such as self-rated adherence, or indirectly through reporting on changes in objective clinical parameters which should improve with adherence promoting interventions.

Fourteen out of the 22 RCTs used both direct measures of adherence e.g., questionnaires, self-reporting or pill counting (pills consumed/pills prescribed), and indirect measures through changes in clinically relevant parameters e.g., systolic blood pressure, HbA1c, or health-related quality of life as end-outcomes (44, 46, 47, 49, 50, 52–54, 57, 59, 60, 63, 64, 66). Four studies only used indirect measures through changes in clinically relevant parameters (51, 58, 62, 65) and three studies only used direct measures of subjective adherence (48, 55, 56). One study used both objective and subjective direct measures of adherence (45).

Of the 18 studies that used direct measures of adherence as end-outcomes, three used objective measures of adherence e.g., pill counting (all three studies) (45, 60, 66) and 15 used subjective measures of adherence, such as self-reporting (five studies) (45, 47, 50, 55, 63)or questionnaires (ten studies) (44, 48, 51–54, 56, 57, 59, 64). Questionnaires measuring adherence included the Beliefs about Medicines Questionnaire (BMQ), Morisky Medication Adherence Questionnaire (MMAQ), Medication Adherence Rating Scale (MARS), Medication Adherence Questionnaire (MAQ), and questionnaires specifically designed for the purposes of the relevant study. One study's-outcome was the number of patients advised to adhere to medications rather than medication adherence *per se* (46).

### Risk of bias

**Figure 2:**
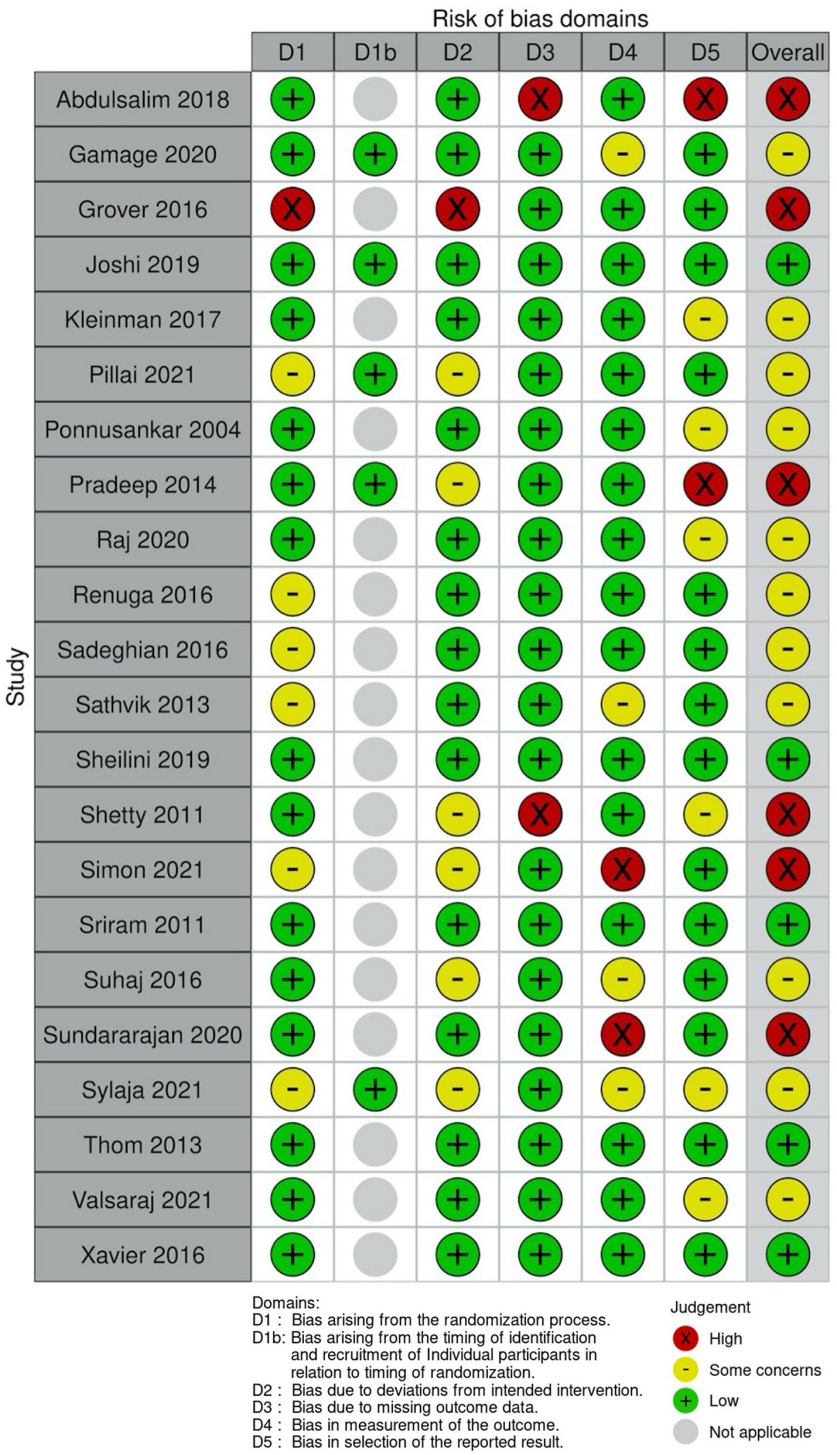
Risk of bias for individual studies.

**Figure 3:**
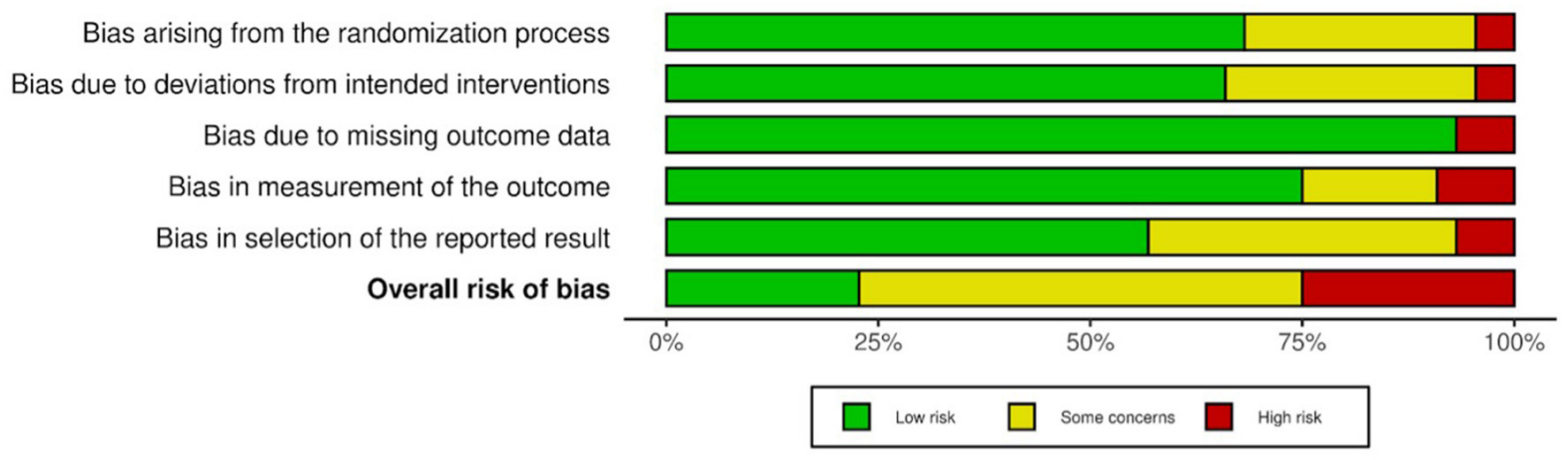
Overall risk of bias.

Out of 22 included studies, five were low risk of bias, (50, 52, 64–66) six were high risk (44, 49, 51, 54, 56, 57) and 11 were assessed as some concerns (14, 45–48, 53, 55, 58, 59, 62, 63) (Figure 2). Notably, two studies were deemed “high risk” and eight assessed as “some concerns” for bias in selection of reported results category (Figure 2). One study was deemed “high risk” and six studies were deemed “some concerns” for bias arising in the randomization process. This was primarily due to lack of reporting of how randomization occurred. One study was assessed as “high risk” while eight were assessed as “some concerns” for bias arising from deviations from intended interventions, mainly due to lack of information surrounding if and how participants and observers were blinded. We also found that only 12 out of 22 studies had registered their study protocol (47, 50, 52, 53, 55–57, 61–64, 66). Overall Risk of Bias is shown in Figure 3. Information on individual study blinding is shown in the Supplementary material.

### Education-based interventions delivered by pharmacists

Seven studies examined educational programmes delivered by pharmacists to improve adherence and all showed significant improvements in adherence outcomes assessed (44, 45, 48, 54, 57, 59, 65). The interventions were generally short (10–30 min), personalized sessions which could be delivered repeatedly, and were often combined with provision of written materials and lifestyle advice. Studies found improvement in both objective and subjective metrics of adherence utilizing different regimens of pharmacist led sessions. All identified studies showed some benefits although some studies (44, 45) did not conduct complete statistical tests of significance (44, 45) whilst others only followed patients up over 2 months (45, 48). All studies except one (57) were based at single centers and all had relatively low sample sizes.

For example, Sriram et al. found that pharmacist teaching comprising education, medication counseling, and lifestyle advice during baseline and follow-up visits led to an improvement in HbA1c values (*p* < 0.01) and diabetes-dependent quality of life (*p* < 0.01) after 8 months compared to usual care across 120 patients with type 2 diabetes (65). Similarly, Simon et al. also found that a verbal counseling session and leaflets from clinical pharmacists focussing on medication adherence and lifestyle advice led to improved HbA1c (*p* < 0.001) and medication adherence (*p* < 0.001) scores compared to conventional therapy with basic counseling across 97 patients with type 2 diabetes after 6 months (57).

These benefits were not limited to type 2 diabetes, with Sundararajan et al. utilizing a single 30-min pharmacist counseling session at discharge, addressing subjects such as adherence and lifestyle advice, in patients post-myocardial infarction (54). They found improved medication adherence (*p* = 0.0001) and cardiovascular clinical parameters such as blood pressure (*p* < 0.01) after 6 months compared to usual care across 154 patients. Further, Sathvik et al. found improved medication adherence scores across belief and recall domains (*p* < 0.05) in hypertensive patients given 4 pharmacist education sessions compared to control patients given leaflets alone in a cohort of 150 over 2 months (48). Ponnusankar et al. was the only study to measure adherence using an objective pill-count method and found improved adherence after 2 months in a group of 90 patients with various chronic diseases following pharmacist a counseling session compared to usual care (45). They found an increase in pill-count scores and self-assessed compliance although they did not carry out formal statistical analyses.

Renuga et al. demonstrated potential added value of repeated interventions, showing that verbal counseling and education leaflets provided at baseline and during 3 follow-ups led to further improvements in adherence (*p* < 0.001) and fasting blood sugar level (*p* < 0.01) for patients with diabetes after 3 months, compared to the improvements seen with counseling at baseline and follow up alone, across 400 patients with type 2 diabetes (59).

Uniquely, Grover et al. looked at a pediatric asthma cohort and found that a structured programme delivered to both parents and children focussing on themes such as asthma knowledge and medication use improved medication adherence to asthmatic medications and asthma control (*p* < 0.01) over 6 months compared to control given an information pack and usual care (44). However, their sample size was only 40 parent-child pairs and no statistical analysis on adherence scores were conducted.

### Educational-based interventions delivered by community healthcare workers

Four studies examined education-based interventions delivered by community healthcare workers (CHWs), non-physicians who are able to perform certain health-related interventions, especially in more rural areas of India (49, 63, 64, 66). These individuals usually received training on how to deliver educational interventions and then visited patients, providing individualized advice, counseling, and encouragement to take medications. Three studies found improvement in adherence parameters, however in two, objective clinical parameters failed to reflect improvements in subjective adherence metrics (49, 66). One study found improved clinical parameters but failed to show improvement in adherence metrics (63). Three of these four studies were large multicentre studies focussing on cardiovascular disease.

Xavier et al. showed the benefits of CHWs in improving both adherence measures and corresponding clinical parameters; in a large multicentre trial they found CHWs were effective at increasing adherence to secondary prevention drugs (*p* = 0.006) for 806 patients with acute coronary syndrome and improved clinical cardiovascular parameters such as systolic blood pressure (*p* = 0.002) after 1 year through providing repeated counseling on lifestyle measures and education (64).

Gamage et al. conducted a large cluster trial with 1,734 participants with hypertension and found trained CHWs significantly improved hypertension control compared to usual care (*p* = 0.001) in 3 socioeconomically distinct regions through their delivery of 6 fortnightly sessions over 3 months to educate patients on their disease, medication, and lifestyle choices (63). However, they did not find significant changes in antihypertensive medication use, indicating this effect may have been primarily due to lifestyle changes.

Joshi et al. (a large cluster trial with 2,312 patients) and Pradeep et al. (randomized trial with 260 women in 6 rural villages) found CHWs were effective at improving adherence for cardiovascular risk factors (through 6 household visits over 12 months in which they ascertained and reinforced, *p* = 0.001) and depression (through twice monthly visits in which education and counseling on medication use was provided for 6 months, *p* < 0.01) compared to usual care respectively, but both did not find significant improvements in corresponding clinical parameters (49, 66). Joshi et al. also looked at adherence and BP 6 months after the intervention ended and found a rise in mean blood pressure and a drop in adherence (61).

### Educational-based interventions delivered by a multidisciplinary team

Sadeghian et al. (314 patients across two hospitals) utilized a self-management education programme delivered for 2 hours a week over 2 weeks, by a multidisciplinary team to patients with T2DM (58). The intervention team consisted of an endocrinologist, diabetologist, public health expert, dietician, diabetes nurse educator and the investigator. They reported an objective improvement in HbA1c at the 6-months follow-up period (*p* < 0.001).

### Training practitioners

Sylaja et al. conducted a cluster RCT of 243 subjects investigating the effects of improved CHW competency on the secondary prevention of stroke over a period of six months (46). Their intervention consisted of four 45-min training sessions for CHWs focused on the management of acute stroke, drug adherence, care, and physiotherapy. At 6 months follow-up there was an increase in the number of patients advised on medication adherence by CHWs in the intervention group compared to CHWs in the control group (*p* < 0.001), but this was not associated with significant improvements in blood pressure or fasting blood sugar in the intervention group.

### Combining patient education with regular follow-up

Four studies (based on 3 RCTs) focused on multimodal interventions by non-physicians, providing education and counseling in combination with regular follow-up interventions (52, 56, 60, 62). All showed improvements in medication adherence metrics although one did not measure clinical parameters (56) although two showed no improvement in clinical outcome measures (52, 60).

Abdulsalim et al. conducted a single-center RCT looking at the benefits pharmacist-led counseling for 260 patients with COPD over a period of 2 years, in combination with monthly telephone calls, to ensure adherence (56). Pharmacist sessions focused on medication adherence and education, as well as lifestyle information, and leaflets were also provided. They found significant improvements in subjectively rated medication adherence (*p* < 0.001) after 2 years. The study was based on the results of a study by Suhaj et al. who independently reported significant improvement in health-related quality of life (*p* < 0.001) in the same cohort (62).

Sheilini et al. conducted a single-center RCT, investigating the benefits of a nurse-led intervention for 160 hypertensive patients non-adherent to medications over a period of 6 months (52). The intervention involved personalized education, information leaflets in combination with weekly reminder boxes and a single telephone follow-up. They reported improved adherence (*p* < 0.001) but no significant reduction in blood pressure (*p* = 0.16) for the intervention group compared to controls, which may have been due to unmodified lifestyle factors.

Raj et al. looked at tailored advice delivered by a study investigator (such as education if there was poor knowledge) combined with diary and mobile phone reminders in 50 older patients with a range of NCDs (60). They found a significant increase in reported pill counts after 3 months (*p* = 0.007) and 6 months (*p* = 0.003) although no significant changes in corresponding clinical parameters. However, they found a decrease in reported pill counts at 6 months relative to 3 months (*p* = 0.016).

### Technology-based interventions

Two studies investigated the effects of reminder-based systems on medication adherence (47, 51).

Kleinman et al. assessed the impact of a Gather Health, a mobile-Health diabetes management platform on medication adherence and frequency of blood glucose self-testing at three centers across 91 patients with type 2 diabetes (47). After 6 months follow-up, they found that the intervention group had significantly improved self-reported medication (*p* = 0.03), increased frequency of blood glucose self-testing (*p* = 0.01) and improvement in HbA1c compared to control (*p* = 0.02). Similarly, Shetty et al. conducted a single-center pilot study to investigate the effectiveness of mobile short message services on adherence in 215 diabetic patients (51). Messages were sent once every 3 days, and both content and frequency varied based on subjects” preferences. At 1 year follow-up they reported no significant difference in mean HbA1c values between both groups (*p* value not stated) but found a significant increase in the percentage of subjects with HbA1c < 8% in the intervention group compared to controls (*p* < 0.007). They stated that drug prescriptions were followed satisfactorily by both groups although which specific questionnaire was used was not stated.

### Fixed dose combinations

Thom et al. conducted a large multi-center trial across 1,000 patients investigating the effects of fixed-dose-combination based strategies (polypills) on self-reported adherence and changes in blood pressure in those with cardiovascular disease or high risk of cardiovascular disease (the UMPIRE trial) (50). At follow-up (median = 15 months) those in the intervention group taking a polypill with their CVD medications were found to have improved self-reported medication adherence compared to usual care (*p* < 0.001). Improvements in adherence were associated with improvements in clinical parameters such as reductions in systolic blood pressure (*p* < 0.001) and LDL-C (*p* < 0.001).

### Psychological therapies

Valsaraj et al. found that weekly 50-min cognitive behavioral therapy (CBT) sessions for 80 patients with chronic kidney disease undergoing dialysis, were associated with increases in dialysis adherence (*p* = 0.001) and drug adherence (*p* < 0.001) compared to non-directive counseling (53). These changes in adherence were associated with concomitant changes in objective parameters such as systolic blood pressure (*p* = 0.001), diastolic blood pressure (*p* = 0.001), Hb (p =0.001), and inter-dialysis weight gain (*p* = 0.001).

Pillai et al. reported on a collaborative stepped care intervention to improve adherence to anti-depressants across 2,796 patients with depression (55). The intervention involved psychoeducation, antidepressants prescribing, and interpersonal psychotherapy delivered according to illness severity and patient response. Compared to normal care (which included increased access antidepressant prescribing), they found improved self-reported adherence over a course of a month (OR = 6.10, 95% CI 3.67–10.14) and a significantly higher proportion completed at least 90 days of antidepressants, compared to usual care (although exact *p* values are not stated).

### Results by NCDs

Table 3 shows a summary of the evidence for adherence promoting measures broken down by disease. Of the studies retrieved, three studies examined respiratory disease, six focussed on type 2 diabetes, eight on cardiovascular disease, two on depression, one on renal disease and 2 looked across NCDs (not included in Table 3).

**Table 3:** Evidence for adherence promoting interventions broken down by NCD.

**Interventions promoting adherence**	**Strength of evidence**	**Advantages**	**Limitations**
**Respiratory disease**			
Pharmacist led educational sessions combined with follow-up calls to ensure adherence.	1 trial (reported in 2 studies) showing evidence for improved adherence and quality of life in COPD patients.	Improvements in adherence and health-related quality of life. Personalized approach possible. Long follow up period (2 years).	Limited cohort (*n =* 260). No evidence of objective clinical improvements or increased adherence. High risk of methodological bias.
Pharmacist educational programmes for parents and children	1 trial showing evidence for improved asthma control and adherence in pediatric cohorts.	Educational interventions can be beneficial beyond adult populations. Benefits from multi-component, individualized educational interventions from allied-health professionals.	Very limited cohort (*n =* 40). Moderate follow-up (6 months). No objective clinical improvements found. High risk of methodological bias.
**T2DM**			
Pharmacist patient education	Three trials showed evidence for increased adherence and improved HbA1c and fasting blood glucose after 3–8 months.	Improvements in corresponding subjective adherence and objective clinical parameters. Benefits from both one-off and follow-up interventions seen.	Limited sample sizes (mean = 205). Short follow-up period (mean = 4.75 months). Mixed methodological quality.
Reminder-based systems	Two trials that found improvements in medication adherence and HbA1c after 6 month-1 year.	Versatile, cheap programmes based on apps and text messages. Improvements in corresponding subjective adherence and objective clinical parameters.	Limited sample sizes (mean = 153). Moderate follow-up (mean 9 months). Low-mixed methodological quality. Results may not be applicable to those without smartphones or less familiar with technology.
MDT-led group self-management educational programme	One trial showing improvements in HbA1c after 6 months.	MDT-approach can provide guidance on adherence as well as lifestyle advice (across exercise, medication use, diet etc.).	Moderate sample size (*n =* 306) Moderate follow-up (6 months). No evidence of improved adherence measures. Mixed methodological quality. Time and resource intensive intervention.
**CVD (including stroke, TIA, hypertension)**			
Pharmacist patient education	2 trials looking at hypertension and post-MI finding improvements adherence and CVD clinical parameters over 2–6 months.	Improvements in corresponding adherence and objective clinical parameters. Benefits from one off and follow-up sessions.	Limited sample size (mean = 76). Short follow up (mean = 4 months). Mixed methodological quality.
Patient education by CHW	3 trials looking at hypertension, wider CVD risk factors and ACS patients over 3 mo-1 year with improvements in adherence and clinical parameters.	Improvements in adherence and clinical parameters. Large sample size across multiple regions (mean = 1,618). CHWs can also provide lifestyle advice to improve outcomes. Mixed-high methodological quality.	Moderate follow up (mean = 9 months). Repeated visits required. Mixed improvements in corresponding parameters in individual studies—some effects from lifestyle improvements and need to ensure corresponding optimal pharmacological management.
Improved CHW training	One trial looked at improved training for CHWs over 6 months and found no significant improvements in clinical parameters.	Improvements in the number of patients advised to adhere.	Limited cohort (*n =* 234). Patient adherence was not measured explicitly. Moderate follow up (6 months). Mixed methodological quality.
Patient education and follow-up by nurses	One trial looked at nurse teaching, information leaflets and weekly reminders over 6 months and found improvements in adherence	Good methodological quality and improved adherence parameters.	Limited sample size (*n =* 160). Moderate follow up (6 months). No improvements in clinical outcomes.
Fixed dose combination strategies	One trial looked at polypill over 15 months for CVD and found improved adherence and clinical outcomes	Corresponding changes in adherence and clinical parameters. Low cost. Long follow-up (15 months). Large sample size (*n =* 1000). Good methodological quality.	Only beneficial for those requiring multiple medications.
**Psychiatry**			
Collaborative stepped care model including psychotherapy	1 trial looked at a combination of psychoeducation, interpersonal psychotherapy and collaborative care management for depression and found improved adherence after 1 month.	Large sample size (*n =* 2,796). Improved adherence and treatment completion. Many modalities available to improve adherence in stepped fashion for patients with more severe disease.	Short follow up (1 month-90 days). Objective clinical outcomes not assessed. Resource intensive. Moderate methodological quality.
CHW education	One trial looking at CHW education and follow-up showing improved adherence over 6 months.	Improved adherence in rural women with major depression	Sample size (*n =* 250). Moderate follow-up (6 months). No improvements in objective clinical parameters. Poor methodological quality. Need to combine with psychosocial interventions.
**Renal**			
CBT	One trial showing improvements in haemodialysis-related clinical parameters, haemodialysis adherence and drug adherence over 6 months in CKD patients.	Improvements in adherence and clinical parameters. Reduced feeling of hopelessness associated with dialysis.	Limited cohort size (*n =* 80) Moderate follow-up (6 months). High cost and less scalable. Mixed methodological quality.

CBT, cognitive behavioral therapy; CHW, community health worker; COPD, chronic obstructive pulmonary disease; CVD, cardiovascular disease; T2DM, type 2 diabetes mellitus; TIA, transient ischaemic attack.

## Discussion

This review summarizes the evidence from 22 RCTs collectively evaluating adherence strategies in over 10,000 patients with NCDs across India and finds moderate evidence for adherence-improving strategies for patients that improve health outcomes. To our knowledge, it is the first review that systematically evaluates the evidence for adherence promoting interventions in India, focussing on patients with NCDs. There is the most consistent evidence for patient education from CHWs and pharmacists, with further benefits from reminder-based systems. Available evidence is primarily focussed on patients diagnosed with CVD and T2DM.

Eleven studies found that CHWs and pharmacist education was effective at improving adherence, across direct and indirect metrics, which is consistent with lack of knowledge, negative attitudes, and low health literacy as key barriers to adherence in LMICs such as India (14, 67, 68). The efficacy of such interventions is in keeping with the “Information-Motivation-Strategy” model in which it is vital patients have the correct information and believe in their treatment to improve adherence (69). Educational interventions are among the most common strategies evaluated to improve adherence in existing literature, tending also to focus on non-physician health workers such as nurses or pharmacists, and there is moderate evidence of their efficacy from systematic reviews primarily based on MEDCs (20, 21, 70).

CHWs can be trained in large numbers relatively cheaply. They therefore represent potentially cost-effective strategies to improve, adherence, treatment capacity and health-related lifestyle choices and consequently improve health-outcomes (64, 71). This is in keeping with existing literature, where similar “lay health workers” have been found to be effective and scalable interventions in the context of communicable diseases globally with the potential to reduce costs of healthcare through task-shifting (72). Both a recent WHO review of reviews focussing predominantly on LMICs, and other LMIC specific systematic reviews, have also found that CHWs can improve outcomes and reduce healthcare costs through a variety of health-related tasks such as screening and counseling as well as adherence-promotion, although common limitations cited also include mixed methodological quality and study heterogeneity (66, 71, 73, 74). Furthermore, health-systems level changes such as allowing CHWs to provide medications directly to patients, can help overcome access and cost barriers and thus provide further benefits (66, 75). CHWs can reach individuals who are less likely to access services such as pharmacists or inpatient facilities and provide a personalized approach to address heterogenous health barriers that exist for such individuals (63, 66). Therefore, a combination of health-education providers, involving doctors, pharmacists and CHWs, may be necessary in India to reach individuals in rural and remote settings, balancing efficacy with increased costs of more time-consuming individual based interventions.

Four studies found that reminder-based systems, in the form of either telephone calls or text messages, increased adherence when combined with education strategies (52, 56, 60, 62). Their findings are in concordance with available literature; a review focussing on CVD across LMICs also found benefits when education was combined with reminders (22); reviews from LMICs focussing on antiretroviral therapy found additive benefits from multimodal interventions involving electronic reminders combined measures such as counseling (24, 76); and a large review, predominantly including MEDC-based studies, also found an additional benefit of reminder-based systems when combined with educational initiatives (20). However, the effects of reminder-based systems when used alone were ambiguous (47, 51), perhaps reflecting the need to first address underlying barriers to adherence attributable to lack of patient education or suboptimal patient provider communication prior to addressing patient forgetfulness (14). Several meta-analyses conducted in MEDCs have found mobile-based reminders to be effective at increasing medication adherence for T2DM and cardiovascular disease through overcoming practical barriers of adherence and supporting disease self-management (77, 78). Reminder-based interventions when used in isolation may be less effective in India compared to MEDCs due to lower rates of health literacy and smartphone usage, both of which are rapidly increasing (79–81).

More resource intensive interventions such as MDT-mediated patient education and psychological based therapies e.g., CBT and psychoeducation were found to be effective at increasing medication adherence (53, 55, 58). The benefits of psychosocial interventions in improving adherence have also been shown in a systematic review for patients with bipolar disorder in LMICs (23). Given the difficulty in providing large-scale initiatives through MDT- and psychological-based approaches, given their relative time and resource demands such interventions may best be used selectively with those with poor adherence refractory to other interventions.

One study found that use of a FDCs increased medication adherence for CVD (50). This was in keeping with a large review from LMICs focussing on CVD which found FDCs to be the most effective strategy for improving medication adherence (22) and a large systematic review of 67 studies, mostly conducted in MEDCs, which also found FDCs to improve adherence compared to multiple pill regimens (82). Whilst FDCs reduces choice of medications and doses, risking suboptimal control of CVD clinical parameters, the authors found this was offset by improved adherence through reducing medication-regimen complexity (12, 50). As FDCs represent promising adherence promoting strategies, further studies are needed to assess their use for treating a larger variety of NCDs that typically require polypharmacy, such as diabetes and CVD, along with public health measures to address accessibility and affordability barriers (83).

### Measures of adherence

Several studies found significant improvements in subjective adherence metrics without corresponding changes in objective clinical parameters (46, 49, 52, 60, 66). This may have reflected inadequate pharmacological therapy, emphasizing the importance of optimizing medical management, such as dose titration or additions of second line medications, in combination with adherence-promoting interventions (66, 84–86). It may also reflect trials that did not assess concomitant lifestyle changes alongside improving medication adherence; the potential for CHWs and pharmacists to counsel on weight loss, exercise and smoking cessation represent effective additional methods of improving health outcomes (52). Finally, small study sizes, limited time frames and controls which encouraged patients to seek additional care may also be responsible for a proportion of this effect (49, 60). We recommend future interventions aiming to improve medication utilize regular reviews to optimize a patients' pharmacological management and involve simultaneous counseling and assessment of lifestyle measures.

### Limitations

This review's conclusions are limited by mixed methodological quality in included studies. Moderate and high risk of bias across studies was primarily due to bias in selection of reporting results, lack of information on how randomization occurred, and if and how participants and observers were blinded. To mitigate reporting bias, we recommended that future RCTs pre-register their study protocol online. Additionally, more information is required on randomization and blinding processes used to ensure high methodological quality.

Additional limitations relate to the nature of the included studies. Firstly, there is volunteer bias to which all RCTs are subject, meaning it is unclear how generalizable these findings are to those who are more vulnerable or with lower health literacy. Secondly, most included studies primarily used subjective measures of adherence such as validated questionnaires or self-reporting, while only three employed objective measures of adherence. Subjective measures are liable to recall bias and social desirability bias, generally resulting in over-reporting and may limit the generalizability of our results, although a range of these questionnaires have been found to be valid across NCDs (87–90). Thirdly, of the available studies, there were few large multicentre RCTs and in general follow-up periods were short (median follow-up was 6 months). As medication adherence rates tend to decline with time from intervention possibly due to participant fatigue and waning novelty, follow-up mechanisms and evaluating for sustained effects are crucial to determining the efficacy of adherence promoting measures (24, 60, 66). Fourthly, given the relatively small number of included studies (22) across a large number of states and regions within India it was not possible to give a breakdown by state, where different healthcare systems and socioeconomic factors may influence adherence barriers and interventions. Finally, the COVID-19 pandemic has driven a shift that has rapidly changed the field of medication adherence, so previous studies (15/22 were published before 2020) may lack current day validity, especially for interventions dependent on technology such as mHealth (91).

Future large RCTs with long follow-up periods to test for sustained improvements in adherence, utilizing methods that attenuate volunteer bias (for example methods that increase recruitment rates), are required to further evaluate adherence-improving measures (92, 93). We recommend future studies evaluate adherence-improving measures using a variety of corresponding adherence and clinical metrics, utilizing objective measures of adherence where possible alongside simpler subjective measures.

## Conclusion

We identified 22 RCTs evaluating interventions to improve medication adherence for community-based patients with NCDs in India. Most RCTs used subjective measures of adherence, many of which also measured objective corresponding clinical parameters. The lack of widespread use of and improvements in objective measures of adherence limits the internal validity and generalizability of findings. Furthermore, scalability of these interventions and their incorporation in wider health programmes requires systematic evaluation of real-world efficacy. This is especially relevant for patients from lower socioeconomic backgrounds and for those living in remote regions with limited access to weaker public health systems. Although the majority of primary studies supporting the conclusions were of mixed methodological quality, patient education by CHWs and pharmacists represent promising interventions to improve medication adherence, with further benefits from regular follow-up. Given the rising burden of NCDs and concomitant poor medication adherence, such programmes merit further evaluation as potentially cost-effective and scalable ways to improve health outcomes and quality of life across patients with NCDs in India.

## Data availability statement

The original contributions presented in the study are included in the article/Supplementary material, further inquiries can be directed to the corresponding authors.

## Author contributions

AT: methodology, validation, investigation, writing—original draft, writing—review and editing, supervision, and project administration. RH: methodology, validation, investigation, data curation, writing—original draft, writing—review and editing, and visualization. RS and KG: methodology, validation, investigation, and writing—review and editing. RK and BS: validation, investigation, and writing—review and editing. SB: conceptualization, methodology, validation, writing—review and editing, and supervision. All authors contributed to the article and approved the submitted version.

## References

[1] Noncommunicable diseases. Available online at: https://www.who.int/news-room/fact-sheets/detail/noncommunicable-diseases (accessed March 12, 2023).

[2] Menon GR Yadav J John D. Burden of non-communicable diseases and its associated economic costs in India. Soc Sci Humanities Open. (2022) 5:100256. 10.1016/j.ssaho.2022.100256

[3] Han E Suh DC Lee SM Jang S. The impact of medication adherence on health outcomes for chronic metabolic diseases: a retrospective cohort study. Res Social Adm Pharm. (2014) 10:e87–98. 10.1016/j.sapharm.2014.02.001 25088545

[4] Tesfaye WH McKercher C Peterson GM Castelino RL Jose M Zaidi STR . Medication Adherence, Burden and Health-Related Quality of Life in Adults with Predialysis Chronic Kidney Disease: A Prospective Cohort Study. Int J Environ Res Public Health. (2020) 17:371. 10.3390/ijerph17010371 31935851 PMC6981524

[5] E. S. Adherence to Long-term Therapies - Evidence for Action. Geneva: World Health Organization (2003).

[6] Akeroyd JM Chan WJ Kamal AK Palaniappan L Virani SS. Adherence to cardiovascular medications in the South Asian population: A systematic review of current evidence and future directions. World J Cardiol. (2015) 7:938. 10.4330/wjc.v7.i12.938 26730300 PMC4691821

[7] Sharma D Goel NK Lehl SS Walia DK Puri S Shukla K . Prevalence and predictors of medication nonadherence among hypertensive patients. Int J Noncommun Dis. (2022) 7:71. 10.4103/jncd.jncd_11_22

[8] Venkatesan M Dongre A Ganapathy K. A community-based study on diabetes medication nonadherence and its risk factors in rural Tamil Nadu. Indian J Community Med. (2018) 43:72–6. 10.4103/ijcm.IJCM_261_17 29899603 PMC5974838

[9] Sahoo J Mohanty S Kundu A Epari V Sahoo J Mohanty S . Medication adherence among patients of type ii diabetes mellitus and its associated risk factors: a cross-sectional study in a tertiary care hospital of eastern India. Cureus. (2022) 14:33074. 10.7759/cureus.33074 36721541 PMC9883658

[10] Peh KQE Kwan YH Goh H Ramchandani H Phang JK Lim ZY . An adaptable framework for factors contributing to medication adherence: results from a systematic review of 102 conceptual frameworks. J Gen Intern Med. (2021) 36:2784–95. 10.1007/s11606-021-06648-1 33660211 PMC8390603

[11] Krishnamoorthy Y Rajaa S Rehman T Thulasingam M. Patient and provider's perspective on barriers and facilitators for medication adherence among adult patients with cardiovascular diseases and diabetes mellitus in India: a qualitative evidence synthesis. BMJ Open. (2022) 12:e055226. 10.1136/bmjopen-2021-055226 35332041 PMC8948385

[12] Shani SD Sylaja PN Sankara Sarma P Raman Kutty V. Facilitators and barriers to medication adherence among stroke survivors in India. J Clin Neurosci. (2021) 88:185–90. 10.1016/j.jocn.2021.03.019 33992182

[13] Choudhary R Sharma SM Kumari V Gautam D. Awareness, treatment adherence and risk predictors of uncontrolled hypertension at a tertiary care teaching hospital in Western India. Indian Heart J. (2016) 68:S251–2. 10.1016/j.ihj.2016.08.003 27751307 PMC5067811

[14] Krishnamoorthy Y Giriyappa DK Eliyas SK Priyan S Saya GK Lakshminarayanan S. Patient and provider's experience and perspective in addressing barriers to medication adherence among noncommunicable disease patients in rural Puducherry, South India—A qualitative study. J Patient Exp. (2019) 6:216. 10.1177/2374373518787288 31535010 PMC6739684

[15] Kini V Michael Ho P. Interventions to improve medication adherence: a review. JAMA. (2018) 320:2461–73. 10.1001/jama.2018.19271 30561486

[16] Singh M Yadav K Goswami S Parashar M Gupta E Verma M . Predictors of adherence to prescribed antihypertensive medications among Hypertensive (15-49 years) in India: A secondary data analysis of National Family Health Survey 4. J Family Med Prim Care. (2022) 11:5807. 10.4103/jfmpc.jfmpc_164_22 36505527 PMC9731078

[17] Deichmann RE Morledge MD Ulep R Shaffer JP Davies P Van Driel ML . metaanalysis of interventions to improve adherence to lipid-lowering medication. Cochrane Database Syst Rev. (2016) 12:CD004371. 10.1016/S0735-1097(16)31955-6 28000212 PMC6464006

[18] Sapkota S Brien JE Greenfield JR Aslani P. A Systematic review of interventions addressing adherence to anti-diabetic medications in patients with type 2 diabetes—components of interventions. PLoS ONE. (2015) 10:e0128581. 10.1371/journal.pone.0128581 26053004 PMC4460122

[19] Cross AJ Elliott RA Petrie K Kuruvilla L George J. Interventions for improving medication-taking ability and adherence in older adults prescribed multiple medications. Cochrane Database Syst Rev. (2020) 2020:CD012419. 10.1002/14651858.CD012419.pub2 32383493 PMC7207012

[20] Torres-Robles A Wiecek E Tonin FS Benrimoj SI Fernandez-Llimos F Garcia-Cardenas V. Comparison of interventions to improve long-term medication adherence across different clinical conditions: A systematic review with network meta-analysis. Front Pharmacol. (2018) 9:1454. 10.3389/fphar.2018.01454 30618748 PMC6311651

[21] Anderson LJ Nuckols TK Coles C Le MM Schnipper JL Shane R . A systematic overview of systematic reviews evaluating medication adherence interventions. Am J Health-System Phar. (2020) 77:138–47. 10.1093/ajhp/zxz284 31901098 PMC6946938

[22] Ogungbe O Byiringiro S Adedokun-Afolayan A Seal SM Himmelfarb CRD Davidson PM . Medication adherence interventions for cardiovascular disease in low- and middle-income countries: a systematic review. Patient Prefer Adherence. (2021) 15:885–97. 10.2147/PPA.S296280 33953548 PMC8092634

[23] Arnbjerg CJ Rurangwa NU Musoni-Rwililiza E Gishoma D Carlsson J Kallestrup P. Intervention trials for adults with bipolar disorder in low-income and lower-middle-income countries: A systematic review. J Affect Disord. (2022) 311:256–66. 10.1016/j.jad.2022.05.097 35605708

[24] Kanters S Park JJH Chan K Socias ME Ford N Forrest JI . Interventions to improve adherence to antiretroviral therapy: a systematic review and network meta-analysis. Lancet HIV. (2017) 4:S187–204. 10.1016/S2352-3018(16)30206-5 27863996

[25] Mills EJ Nachega JB Buchan I Orbinski J Attaran A Singh S . Adherence to antiretroviral therapy in sub-saharan Africa and North America: a meta-analysis. JAMA. (2006) 296:679–90. 10.1001/jama.296.6.679 16896111

[26] Lahai M Theobald S Wurie HR Lakoh S Erah PO Samai M . Factors influencing adherence to antiretroviral therapy from the experience of people living with HIV and their healthcare providers in Sierra Leone: a qualitative study. BMC Health Serv Res. (2022) 22:1327. 10.1186/s12913-022-08606-x 36348488 PMC9644013

[27] Ravindranath V Sundarakumar JS. Changing demography and the challenge of dementia in India. Nat Rev Neurol. (2021) 17:747–58. 10.1038/s41582-021-00565-x 34663985 PMC8522537

[28] Chan AHY Cooper V Lycett H Horne R. Practical barriers to medication adherence: what do current self- or observer-reported instruments assess? Front Pharmacol. (2020) 11:1. 10.3389/fphar.2020.00572 32477110 PMC7237632

[29] World Health Organization (WHO). World Health Organization. Package of Essential Noncommunicable (PEN) Disease Intervention for Primary Health Care. (2020). Available online at: https://iris.paho.org/handle/10665.2/52998 (accessed May 05, 2023).

[30] Page MJ McKenzie JE Bossuyt PM Boutron I Hoffmann TC Mulrow CD . The PRISMA 2020 statement: an updated guideline for reporting systematic reviews. BMJ. (2021) 372:71. 10.1136/bmj.n71 33782057 PMC8005924

[31] Cochrane Handbook for Systematic Reviews of Interventions | Cochrane Training. Available online at: https://training.cochrane.org/handbook/current (accessed March 13, 2023).

[32] SAGE, Wave-2 | *International Institute for Population Sciences (IIPS)*,. Available online at: https://iipsindia.ac.in/content/SAGE-wave-2 (accessed March 13, 2023).

[33] Arinaminpathy N Chin DP Sachdeva KS Rao R Rade K Nair SA . Modelling the potential impact of adherence technologies on tuberculosis in India. Int J Tuberculosis Lung Dis. (2020) 24:526–33. 10.5588/ijtld.19.0472 32398203

[34] Santra S Garg S Basu S Sharma N Singh MM Khanna A. The effect of a mhealth intervention on anti-tuberculosis medication adherence in Delhi, India: A quasi-experimental study. Indian J Public Health. (2021) 65:34. 10.4103/ijph.IJPH_879_20 33753687

[35] Saha S Saxena D Raval D Halkarni N Doshi R Joshi M . Tuberculosis Monitoring Encouragement Adherence Drive (TMEAD): Toward improving the adherence of the patients with drug-sensitive tuberculosis in Nashik, Maharashtra. Front Public Health. (2022) 10:1021427. 10.3389/fpubh.2022.1021427 36620234 PMC9812554

[36] Ekstrand ML Heylen E Pereira M D'Souza J Nair S Mazur A . A behavioral adherence intervention improves rates of viral suppression among adherence-challenged people living with HIV in South India. AIDS Behav. (2020) 24:2195. 10.1007/s10461-020-02785-6 31933020 PMC7319881

[37] Cook R Waldrop-Valverde D Sharma A Vamos S Mahajan B Weiss SM . Cognitive functioning, depression, and HIV medication adherence in India: a randomized pilot trial. Health Psychol Behav Med. (2014) 2:640–52. 10.1080/21642850.2014.913487 25750807 PMC4346084

[38] Skou ST Roos EM. Physical therapy for patients with knee and hip osteoarthritis: supervised, active treatment is current best practice. Clin Exp Rheumatol. (2019) 37:112–7. 31621559

[39] Bannuru RR Osani MC Vaysbrot EE Arden NK Bennell K Bierma-Zeinstra SMA . OARSI guidelines for the non-surgical management of knee, hip, and polyarticular osteoarthritis. Osteoarthritis Cartilage. (2019) 27:1578–89. 10.1016/j.joca.2019.06.011 31278997

[40] Kwon Y Lemieux M Mctavish J Wathen N. Identifying and removing duplicate records from systematic review searches*. 26512216 10.3163/1536-5050.103.4.004 PMC4613377

[41] Ouzzani M Hammady H Fedorowicz Z Elmagarmid A. Rayyan-a web and mobile app for systematic reviews. Syst Rev. (2016) 5:210. 10.1186/s13643-016-0384-4 27919275 PMC5139140

[42] Higgins JP Savović J Page MJ Sterne JAC. RoB 2 Guidance: Parallel Trial. In: The Cochrane Collaboration. (2019) 1–24.

[43] RStudio Team (2020). RStudio: Integrated Development for R. Available online at: http://www.rstudio.com (accessed March 13, 2023).

[44] Grover C Goel N Armour C Van Asperen PP Gaur SN Moles RJ . Medication education program for Indian children with asthma: A feasibility study. Niger J Clin Pract. (2016) 19:76–84. 10.4103/1119-3077.173716 26755223

[45] Ponnusankar S Surulivelrajan M Anandamoorthy N Suresh B. Assessment of impact of medication counseling on patients” medication knowledge and compliance in an outpatient clinic in South India. Patient Educ Couns. (2004) 54:55–60. 10.1016/S0738-3991(03)00193-9 15210260

[46] Sylaja PN Singh G Sivasambath S Arun K Jeemon P Antony R . Secondary prevention of stroke by a primary health care approach: An open-label cluster randomised trial. J Clin Neurosci. (2021) 84:53–9. 10.1016/j.jocn.2020.12.006 33485600

[47] Kleinman NJ Shah A Shah S Phatak S Viswanathan V. Improved medication adherence and frequency of blood glucose self-testing using an m-health platform versus usual care in a multisite randomized clinical trial among people with type 2 diabetes in India. Telemed J E Health. (2017) 23:733–40. 10.1089/tmj.2016.0265 28328396

[48] Sathvik BS Karibasappa M V Nagavi BG. Self - reported medication adherence pattern of rural Indian patients with hypertension. Asian J Pharmac Clin Res. (2013) 6:49–52.25657510

[49] Pradeep J Isaacs A Shanbag D Selvan S Srinivasan K. Enhanced care by community health workers in improving treatment adherence to antidepressant medication in rural women with major depression. Indian J Med Res. (2014) 139:236–45. 24718398 PMC4001335

[50] Thom S Poulter N Field J Patel A Prabhakaran D Stanton A . Effects of a fixed-dose combination strategy on adherence and risk factors in patients with or at high risk of CVD: the UMPIRE randomized clinical trial. JAMA. (2013) 310:918–29. 10.1001/jama.2013.277064 24002278

[51] Shetty AS Chamukuttan S Nanditha A Raj RKC Ramachandran A. Reinforcement of adherence to prescription recommendations in Asian Indian diabetes patients using short message service (SMS)–a pilot study. J Assoc Physicians India. (2011) 59:711–4. 22616337

[52] Sheilini M Hande HM Prabhu MM Pai MS George A. Impact of multimodal interventions on medication nonadherence among elderly hypertensives: A randomized controlled study. Patient Prefer Adherence. (2019) 13:549–59. 10.2147/PPA.S195446 31114169 PMC6489579

[53] Valsaraj BP Bhat SM Prabhu R Kamath A. Follow-up study on the effect of cognitive behaviour therapy on haemodialysis adherence: a randomised controlled trial. Sultan Qaboos Univ Med J. (2021) 21:e58–e65. 10.18295/squmj.2021.21.01.008 33777424 PMC7968912

[54] Sundararajan S Thukani Sathanantham S Palani S. The effects of clinical pharmacist education on lifestyle modifications of postmyocardial infarction patients in South India: a prospective interventional study. Curr Ther Res Clin Exp. (2020) 92:100577. 10.1016/j.curtheres.2020.100577 32140190 PMC7044637

[55] Pillai A Keyes KM Susser E. Antidepressant prescriptions and adherence in primary care in India: Insights from a cluster randomized control trial. PLoS ONE. (2021) 16:e0248641. 10.1371/journal.pone.0248641 33739982 PMC7978355

[56] Abdulsalim S Unnikrishnan MK Manu MK Alrasheedy AA Godman B Morisky DE. Structured pharmacist-led intervention programme to improve medication adherence in COPD patients: A randomized controlled study. Res Social Adm Pharm. (2018) 14:909–14. 10.1016/j.sapharm.2017.10.008 29104008

[57] Simon MA Raja BY Varughese PC Daniel LM Sowjanya K Kumar JS . Pharmacist led intervention towards management of type 2 diabetes mellitus and assessment of patient satisfaction of care - A prospective, randomized controlled study. In: Diabetes Metabolic Syndrome-Clinical Research Reviews. (2021) p. 15. 10.1016/j.dsx.2021.102208 34298274

[58] Sadeghian HA Madhu SV Agrawal K Kannan AT Agrawal K. Effects of a self-management educational program on metabolic control in type 2 diabetes. Turk J Med Sci. (2016) 46:719–26. 10.3906/sag-1501-115 27513247

[59] Renuga E Ramakrishnan SR Vanitha Rani N Thennarasu P Kannan G. Impact of continuous patient counselling on knowledge, attitude, and practices and medication adherence of diabetic patients attending outpatient pharmacy services. Asian J Pharmac Clin Res. (2016) 9:345–50.

[60] Raj JP Mathews B. Effect of behavioral intervention on medication adherence among elderly with select non-communicable diseases (ENDORSE): Pilot randomized controlled trial. Geriatr Gerontol Int. (2020) 20:1079–84. 10.1111/ggi.14032 32896089

[61] Joshi R Agrawal T Fathima F Usha T Thomas T Misquith D . Cardiovascular risk factor reduction by community health workers in rural India: A cluster randomized trial. Am Heart J. (2019) 216:9–19. 10.1016/j.ahj.2019.06.007 31377568 PMC6842688

[62] Suhaj A Manu MK Unnikrishnan MK Vijayanarayana K Mallikarjuna Rao C. Effectiveness of clinical pharmacist intervention on health-related quality of life in chronic obstructive pulmonary disorder patients - a randomized controlled study. J Clin Pharm Ther. (2016) 41:78–83. 10.1111/jcpt.12353 26775599

[63] Gamage DG Riddell MA Joshi R Thankappan KR Chow CK Oldenburg B . Effectiveness of a scalable group-based education and monitoring program, delivered by health workers, to improve control of hypertension in rural India: A cluster randomised controlled trial. PLoS Med. (2020) 17:1002997. 10.1371/journal.pmed.1002997 31895945 PMC6939905

[64] Xavier D Gupta R Kamath D Sigamani A Devereaux PJ George N . Community health worker-based intervention for adherence to drugs and lifestyle change after acute coronary syndrome: a multicentre, open, randomised controlled trial. Lancet Diabetes Endocrinol. (2016) 4:244–53. 10.1016/S2213-8587(15)00480-5 26857999

[65] Srirama S Chacka LE Ramasamya R Ghasemia A Ravia TK Sabzghabaee AM. Impact of pharmaceutical care on quality of life in patients with type 2 diabetes mellitus. J Res Med Sci. (2011) 16:S412. 22247727 PMC3252774

[66] Joshi R Alim M Kengne AP Jan S Maulik PK Peiris D . Task shifting for non-communicable disease management in low and middle income countries–a systematic review. PLoS ONE. (2014) 9:103754. 10.1371/journal.pone.0103754 25121789 PMC4133198

[67] Bowry ADK Shrank WH Lee JL Stedman M Choudhry NK A. systematic review of adherence to cardiovascular medications in resource-limited settings. J Gen Intern Med. (2011) 26:1479–91. 10.1007/s11606-011-1825-3 21858602 PMC3235604

[68] Chauke GD Nakwafila O Chibi B Sartorius B Mashamba-Thompson T. Factors influencing poor medication adherence amongst patients with chronic disease in low-and-middle-income countries: A systematic scoping review. Heliyon. (2022) 8:e09716. 10.1016/j.heliyon.2022.e09716 35770147 PMC9234585

[69] DiMatte MR Haskard-Zolnierek KB Martin LR. Improving patient adherence: a three-factor model to guide practice. Health Psychol Rev. (2011) 6:74–91. 10.1080/17437199.2010.537592

[70] Ampofo AG Khan E Ibitoye MB. Understanding the role of educational interventions on medication adherence in hypertension: A systematic review and meta-analysis. Heart Lung. (2020) 49:537–47. 10.1016/j.hrtlng.2020.02.039 32127208

[71] Vaughan K Kok MC Witter S Dieleman M. Costs and cost-effectiveness of community health workers: Evidence from a literature review. Hum Resour Health. (2015) 13:1–16. 10.1186/s12960-015-0070-y 26329455 PMC4557864

[72] Lewin S Munabi-Babigumira S Glenton C Daniels K Bosch-Capblanch X van Wyk BE . Lay health workers in primary and community health care for maternal and child health and the management of infectious diseases. Cochr Datab System Rev. (2010) 2017:CD004015. 10.1002/14651858.CD004015.pub3 20238326 PMC6485809

[73] Mbuthia GW Magutah K Pellowski J. Approaches and outcomes of community health worker's interventions for hypertension management and control in low-income and middle-income countries: systematic review. BMJ Open. (2022) 12:e053455. 10.1136/bmjopen-2021-053455 35365519 PMC8977767

[74] What do we know about community health workers? A systematic review of existing reviews. Available online at: https://www.who.int/publications/i/item/what-do-we-know-about-community-health-workers-a-systematic-review-of-existing-reviews (accessed March 13, 2023).

[75] Heller DJ Kumar A Kishore SP Horowitz CR Joshi R Vedanthan R. Assessment of barriers and facilitators to the delivery of care for noncommunicable diseases by nonphysician health workers in low- and middle-income countries: a systematic review and qualitative analysis. JAMA Netw Open. (2019) 2:e1916545–e1916545. 10.1001/jamanetworkopen.2019.16545 31790570 PMC6902752

[76] Mills EJ Lester R Thorlund K Lorenzi M Muldoon K Kanters S . Interventions to promote adherence to antiretroviral therapy in Africa: a network meta-analysis. Lancet HIV. (2014) 1:e104–11. 10.1016/S2352-3018(14)00003-4 26424119 PMC5096455

[77] Haider R Sudini L Chow CK Cheung NW. Mobile phone text messaging in improving glycaemic control for patients with type 2 diabetes mellitus: A systematic review and meta-analysis. Diab Res Clin Pract. (2019) 150:27–37. 10.1016/j.diabres.2019.02.022 30822496

[78] Shariful Islam SM Farmer AJ Bobrow K Maddison R Whittaker R Pfaeffli Dale LA . Mobile phone text-messaging interventions aimed to prevent cardiovascular diseases (Text2PreventCVD): systematic review and individual patient data meta-analysis. Open Heart. (2019) 6:1017. 10.1136/openhrt-2019-001017 31673381 PMC6802999

[79] India Literacy Rate 1981-2023 | MacroTrends. Available online at: https://www.macrotrends.net/countries/IND/india/literacy-rate (accessed March 13, 2023).

[80] D AM Shankar Aradhya MR. Health literacy among Indian adults seeking dental care. Dent Res J. (2013) 10:20–4. 10.4103/2319-5932.167618 23878559 PMC3714819

[81] India: smartphone penetration rate 2040 | Statista. Available online at: https://www.statista.com/statistics/1229799/india-smartphone-penetration-rate/ (accessed March 13, 2023).

[82] Baumgartner A Drame K Geutjens S Airaksinen M. Does the Polypill Improve Patient Adherence Compared to Its Individual Formulations? A Systematic Review. Pharmaceutics. (2020) 12:190. 10.3390/pharmaceutics12020190 32098393 PMC7076630

[83] Webster R Murphy A Bygrave H Ansbro É Grobbee DE Perel P. Implementing fixed dose combination medications for the prevention and control of cardiovascular diseases. Glob Heart. (2020) 15:860. 10.5334/gh.860 32923350 PMC7442173

[84] Datta S. Utilization study of antihypertensives in a south indian tertiary care teaching hospital and adherence to standard treatment guidelines. J Basic Clin Pharm. (2016) 8:33. 10.4103/0976-0105.195100 28104972 PMC5201061

[85] Birhanu MM Evans RG Zengin A Riddell M Kalyanram K Kartik K . Absolute cardiovascular risk scores and medication use in rural India: a cross-sectional study. BMJ Open. (2022) 12:e054617. 10.1136/bmjopen-2021-054617 35459666 PMC9036467

[86] Mehdi S Manohar K Shariff A Wani SUD Almuqbil M Alshehri S . Analysis of antidepressants utilization for patients visiting psychiatric out-patient clinic in a tertiary care hospital. Healthcare (Switzerland). (2022) 10:1–13. 10.3390/healthcare10102081 36292530 PMC9602627

[87] Nguyen TMU La Caze A Cottrell N. Validated adherence scales used in a measurement-guided medication management approach to target and tailor a medication adherence intervention: a randomised controlled trial. BMJ Open. (2016) 6:e013375. 10.1136/bmjopen-2016-013375 27903564 PMC5168495

[88] Morisky DE Green LW Levine DM. Concurrent and predictive validity of a self-reported measure of medication adherence. Med Care. (1986) 24:67–74. 10.1097/00005650-198601000-00007 3945130

[89] Thompson K Kulkarni J Sergejew AA. Reliability and validity of a new Medication Adherence Rating Scale (MARS) for the psychoses. Schizophr Res. (2000) 42:241–7. 10.1016/S0920-9964(99)00130-9 10785582

[90] Jimenez K Vargas C Garcia K Guzman H Angulo M Billimek J. Evaluating the validity and reliability of the beliefs about medicines questionnaire in low-income, spanish-speaking patients with diabetes in the United States HHS public access. Diabetes Educ. (2017) 43:114–24. 10.1177/0145721716675740 27831521 PMC5899517

[91] Ruksakulpiwat S Zhou W Niyomyart A Wang T Kudlowitz A. How does the COVID-19 pandemic impact medication adherence of patients with chronic disease? A systematic review. Chronic Illn. (2022) 16:17423953221110151. 10.1177/17423953221110151 35971949 PMC9382573

[92] Patel MX Doku V Tennakoon L. Challenges in recruitment of research participants. Adv Psychiatr Treatment. (2003) 9:229–38. 10.1192/apt.9.3.229

[93] Jordan S Watkins A Storey M Allen SJ Brooks CJ Garaiova I . Volunteer bias in recruitment, retention, and blood sample donation in a randomised controlled trial involving mothers and their children at six months and two years: a longitudinal analysis. PLoS ONE. (2013) 8:67912. 10.1371/journal.pone.0067912 23874465 PMC3706448

