# Supplementary material for: Interventions to promote medication adherence for chronic diseases in India: a systematic review
Source: Front Public Health. 2023 Jun 16;11:1194919. doi: 10.3389/fpubh.2023.1194919 (PMC10311913; doi:10.3389/fpubh.2023.1194919)
Supplement: Supplementary file 1 [file Data_Sheet_1.DOCX]

A systematic review on interventions to promote medication adherence for chronic diseases in India

***Authors:*** *Abraham Tolley* (University of Cambridge), Refaat Hassan* (University of Cambridge), Rohan Sanghera (University of Cambridge), Kirpal Grewal (University of Cambridge), Ruige Kong (University of Cambridge), Baani Sodhi (Indian Institute of Public Health - Delhi), Dr Saurav Basu (Indian Institute of Public Health - Delhi)*

**Equal contribution and first authorship: These authors contributed equally to this work and share first authorship*

***Senior author:*** *Dr Saurav Basu*

***Co-corresponding:*** *Refaat Hassan, Dr Saurav Basu*

# Search Terms

**Outcome: Adherence**

- Patient compliance
- Patient adherence
- Dropout
- Treatment refusal
- Patient education

**Outcome: Medications**

- Medic*
- Drug
- Treatment
- Drug therapy

**Population: Chronic Diseases**

1. Chronic disease
2. Diabetes
3. Chronic lung disease
4. Interstitial lung disease
5. Pulmonary fibrosis
6. COPD (chronic obstructive pulmonary disease)
7. Asthma
8. Cardiovascular disease
9. Angina
10. Hypertension
11. Stroke
12. Cerebrovascular accident
13. Heart disease
14. Myocardial infarction
15. Arrhythmia
16. Peripheral arter* disease
17. Mental health
18. Depression
19. Anxiety
20. Parkinson’s disease
21. Dementia

**Population: India**

- India

# Databases

## Medline via OVID: 331

Ovid MEDLINE(R) and Epub Ahead of Print, In-Process, In-Data-Review & Other Non-Indexed Citations, Daily and Versions <1946 to July 18, 2022>

1 patient compliance.mp. or exp Patient Compliance/ 92380

2 patient adherence.mp. 4526

3 dropout.mp. or exp Patient Dropouts/ 17350

4 treatment refusal.mp. or exp Treatment Refusal/ 13618

5 patient education.mp. or exp Patient Education as Topic/ 106966

6 medic*.mp. 3171164

7 drug.mp. or exp Pharmaceutical Preparations/ 6549229

8 treatment.mp. or exp Therapeutics/ 8820193

9 drug therapy.mp. or exp Drug Therapy/ 3322428

10 6 or 7 or 8 or 9 13964291

11 chronic disease.mp. or exp Chronic Disease/ 627753

12 interstitial lung disease.mp. or exp Lung Diseases, Interstitial/ 86982

13 pulmonary fibrosis.mp. or exp Pulmonary Fibrosis/ 35208

14 copd.mp. or exp Pulmonary Disease, Chronic Obstructive/ 83104

15 exp Asthma/ or asthma.mp. 191420

16 cardiovascular disease.mp. or exp Cardiovascular Diseases/ 2695929

17 exp Angina Pectoris/ or angina.mp. 73266

18 exp Hypertension/ or hypertension.mp. 539822

19 exp Stroke/ or stroke.mp. 369422

20 heart disease.mp. or exp Heart Diseases/ 1296240

21 myocardial infarction.mp. or exp Myocardial Infarction/ 272176

22 arrhythmia.mp. or exp Arrhythmias, Cardiac/ 259578

23 peripheral arter* disease.mp. or exp Peripheral Arterial Disease/ 21334

24 mental health.mp. or exp Mental Health/ 243470

25 exp Depression/ or depression.mp. 463498

26 exp Anxiety/ or anxiety.mp. 286975

27 parkinson's disease.mp. or exp Parkinson Disease/ 119675

28 dementia.mp. or exp Dementia/ 244412

29 diabetes.mp. or exp Diabetes Mellitus/ 748100

30 india.mp. or exp India/ 171990

31 1 or 2 or 3 or 4 or 5 220318

32 chronic lung disease.mp. 7250

33 cerebrovascular accident.mp. 5178

34 11 or 12 or 13 or 14 or 15 or 16 or 17 or 18 or 19 or 20 or 21 or 22 or 23 or 24 or 25 or 26 or 27 or 28 or 29 or 32 or 33 5256845

35 10 and 30 and 31 and 34 331

## Scopus: 683

( TITLE-ABS-KEY ( india ) )  AND  ( TITLE-ABS-KEY ( ( "chronic disease"  OR  diabetes  OR  "chronic lung disease"  OR  "interstitial lung disease"  OR  "pulmonary fibrosis"  OR  copd  OR  asthma  OR  "cardiovascular disease"  OR  angina  OR  hypertension  OR  stroke  OR  "cerebrovascular accident"  OR  "heart disease"  OR  "myocardial infarction"  OR  arrhythmia  OR  "peripheral arter* disease"  OR  "mental health"  OR  depression  OR  anxiety  OR  "parkinson's disease"  OR  dementia ) ) )  AND  ( TITLE-ABS-KEY ( ( "patient compliance"  OR  "patient adherence"  OR  dropout  OR  "treatment refusal"  OR  "patient education" ) ) )  AND  ( TITLE-ABS-KEY ( ( medic*  OR  drug  OR  treatment  OR  "drug therapy" ) ) )

## Web of science: 307

#4 AND #3 AND #2 AND #1

4. ALL=(India)

3. ((((((((((((((((((((ALL=("chronic disease")) OR ALL=(diabetes)) OR ALL=("chronic lung disease")) OR ALL=("interstitial lung disease")) OR ALL=("pulmonary fibrosis")) OR ALL=(COPD)) OR ALL=(asthma)) OR ALL=("cardiovascular disease")) OR ALL=(angina)) OR ALL=(hypertension)) OR ALL=(stroke)) OR ALL =(“cerebrovascular accident”)) OR ALL=(heart disease)) OR ALL=(myocardial infarction)) OR ALL=(arrhythmia)) OR ALL=("peripheral arter* disease")) OR ALL=("mental health")) OR ALL=(depression)) OR ALL=(anxiety)) OR ALL=("parkinson's disease")) OR ALL=(dementia)

2. (((ALL=(medic*)) OR ALL=(drug)) OR ALL=(treatment)) OR ALL=("drug therapy")

1. ((((ALL=("patient compliance")) OR ALL=("patient adherence")) OR ALL=(dropout)) OR ALL=("treatment refusal")) OR ALL=("patient education")

## Google Scholar: 200

Given the limits on Google Scholar search length an abbreviated search strategy was used. The top 200 results were selected for de-duplication and abstract screening.

“India” AND “adherence” OR “compliance” OR “dropout” OR “refusal” OR “education” AND “medication” OR “drug” OR “treatment” OR “therapy”

## Data Extraction Template

The following domains were extracted from each included study

1. Title, authors, year of publication
2. Study design
3. Geographical state/region
4. Disease of interest
5. Details of intervention group and sample size
6. Details of comparison group and sample size
7. Method(s) of assessing adherence and outcome variable(s)
8. Study timeframe
9. Key results and measures of effect size
10. Cost of intervention and cost effectiveness
11. Study advantages
12. Study limitations and disadvantages
13. Other relevant outcomes
14. Additional relevant comments
